# Effects on duration of post-operative ischemia and patterns of blood flow recovery in different conditions of mouse hind limb ischemia

**DOI:** 10.1186/2045-824X-3-14

**Published:** 2011-06-14

**Authors:** Husain A Al-Mubarak, Talal M Alamri, Saif A Aljabab, Mohammad Atteya, Adrian Quan, Hwee Teoh, Praphulla C Shukla, Subodh Verma, Abdullah Aldahmash, Badr Aljabri, Claudio Napoli, Mohammed Al-Omran

**Affiliations:** 1 Peripheral Vascular Disease Research Chair and The Stem Cell Unit, College of Medicine, King Saud University, Riyadh, Saudi Arabia; 2 King Saud University-Li Ka Shing Knowledge Institute of St Michael's Hospital Collaborative Research Program, Toronto, Canada; 3 Department of General Pathology, Division of Clinical Pathology and Excellence Research Center on Cardiovascular Diseases, 1st School of Medicine, II University of Naples, 80138 Naples, Italy

**Keywords:** Hindlimb Ischemia, Angiogenesis, LDPI, Mouse Model, Peripheral Arterial Disease

## Abstract

**Background:**

Current limitations to the experimentation on patients with peripheral arterial disease push the development of different preclinical strategies. We investigated both duration of ischemia and blood flow recovery in mouse models of partial femoral artery ligation.

**Methods:**

Male BALB/c mice were used. The ligation over needle method involved placing a suture needle over the femoral artery, ligating over it and then removing the needle. The transfixation method involved transfixing the approximate center of the femoral artery and then tying the suture. Laser Doppler Perfusion Imaging was used to assess perfusion every 3^rd ^day until 42 days after the procedure.

**Results:**

Ligation over needle method: Immediately post procedure, mean perfusion was -71.87% ± 4.43. Then mean difference in perfusion remained below the base line reading on days 3, 6, 9, and 12. From day 15 on wards mean perfusion progressively improved remaining near base line. Transfixation Method: Immediately post procedure mean perfusion was -70.82% ± 4.73. Mean perfusion improved following the procedure on days 3 and 6; a plateau followed this on days 9, 12 and 15. From day 15 onwards perfusion progressively improved remaining well below base line until crossing it on day 36.

**Conclusion:**

The currently described models do not pose major improvements over previously described methods.

## Introduction

Peripheral arterial disease (PAD) is a clinical manifestation of atherosclerosis affecting the aorto-iliac and infra-inguinal arterial trees. Intermittent claudication is the classical symptom of PAD [1,2]; patients may be asymptomatic or present with non-healing ulcers and tissue loss. More relevantly, PAD can also be considered as a marker of systemic atherosclerosis, as patients with PAD are at greater risk of having myocardial infarction [3] and ischemic stroke [4].

Translational approaches using mouse models of hind limb ischemia have advantages over other animal models. Mice require less food [5], housing space [6], and time to acquire Laser Doppler Perfusion Imaging scans as compared to other rodents.

In general, previously described mouse models of ischemia revolve around the same scheme [7-11]. After exposure, the femoral artery of the hind limb is dissected free from the surrounding tissue. Then ligations are made at various points with or without removing part of the artery with or without the vein. The end result is complete obstruction of the blood supply to the limb. The abrupt and absolute nature of the ischemia induced by these methods is similar to what occurs in acute arterial occlusion. The objective of this study was to develop a murine model of surgically induced ischemia via partial femoral artery ligation that remains ischemic for a minimum period of 27 days.

## Methods and Results

All procedures and protocols were approved by the Institutional Review Committee of The College of Medicine, King Saud University, Riyadh, Saudi Arabia. Animal experiments were performed at the animal housing facilities of The College of Medicine, King Saud University, Riyadh, Saudi Arabia. Male BALB/c mice aged between 11-12 weeks and weighing 23-34 g were used. The animals were anesthetized with Ketamine-Xylazine intraperitonially (IP) for the surgical procedures and for the laser Doppler measurements of limb perfusion.

**Figure 1:**
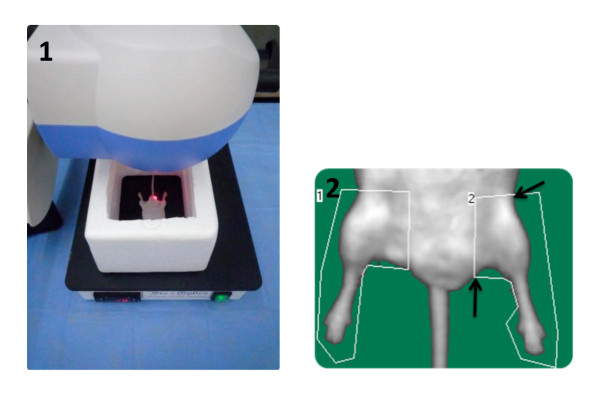
**Positioning and setting used when taking LDPI scans (1), Anatomical landmarks used for determining Regions of interest (2)**.

Laser Doppler perfusion imaging (LDPI) (Perimed, PIM 3) was used to evaluate the perfusion over the course of 6 weeks postoperatively. The mice were placed on a 37°C warming pad, positioned using an alignment triangle and placed between a Styrofoam barrier (See Figure 1). LDPI was used to record perfusion of hind limbs preoperatively, immediately postoperatively (D0) and on every third day following D0. The dimensions of the scanned area and its resolution were kept constant during the entire scanning period, as was the distance of the laser Doppler head from the scanning surface. Serial measurements were obtained over the same region of interest using anatomical landmarks. Analysis was performed by calculating the mean perfusion for both ischemic and non-ischemic limbs from the color coded images. The change in perfusion was expressed as the percentage change between the right (ischemic) to the left (normal, reference) limb. Mice that did not have decrease from baseline measurements immediately post-operatively or on day 3 were excluded from the study. They were considered unsuccessful procedures.

On day 42 of the study, the mice were euthanized with an overdose of anesthetic and cervical dislocation after performing LDPI scans. Histological experiments were done at The Stem Cell Unit, Department of Anatomy, College of Medicine, King Saud University, Riyadh, Saudi Arabia. Samples of skeletal muscle were taken from the calf and thigh of both limbs and placed in 10% formaldehyde till processing. After fixation in paraffin wax, histological sections 5 μm thick were stained with Hematoxylin and Eosin stains and were examined under a light microscope. Skin samples from the medial aspect of both limbs were placed in 10% formaldehyde until fixation in paraffin blocks.

The surgical procedures were performed on the right hind limb. Exposure of the femoral artery was obtained by a skin incision parallel to the inguinal ligament approximately the width of the thigh. After dissecting the proximal portion femoral artery just distal to the inguinal ligament, one of two procedures was performed to induce unilateral hind limb ischemia.

Results are presented as mean ± SE.

### Ligation over needle group

**Figure 2:**
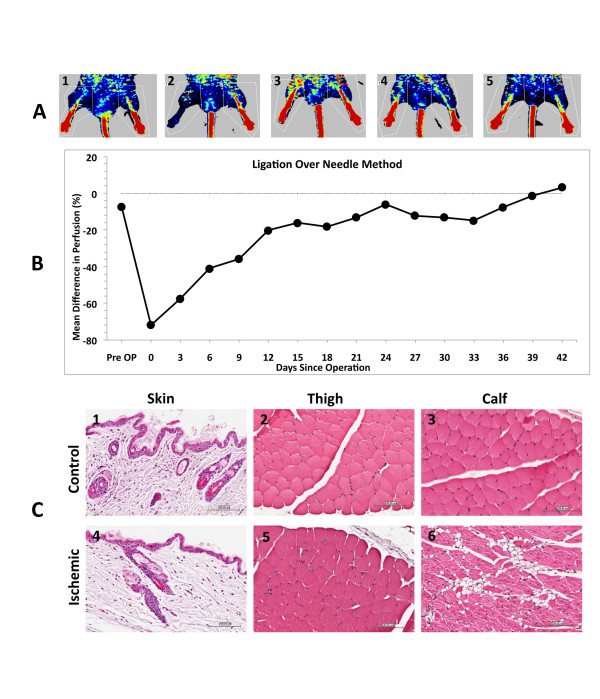
**2A; Laser Doppler perfusion Images, Ligation over needle group; Pre-operative, Post-Operative, Day 12, Day 24 and Day 42 post ischemia (1,2,3,4 and 5 respectively)**. Figure 2B; Mean difference in perfusion (%), Ligation over needle group. Figure 2C; Skin from medial aspect of left thigh with normal appearance of dermis, epidermis and skin appendages (1); Left thigh with normal appearance of skeletal muscle fibers (2); Left calf with normal appearance of skeletal muscle fibers (3); Skin from medial aspect of right thigh with normal appearance of dermis, epidermis and skin appendages (4); Right thigh with normal appearance of skeletal muscle fibers (5); Right calf with vascular congestion, damage to some skeletal muscle fibers and replacement by adipose tissue (6). All sections were taken at 200×, stained with Hematoxylin and Eosin stain. Scale bars are 100 μm.

The ligation over needle method involved placing a 10-0 suture needle (Ethicon) and ligating over it with a 9-0 prolene (Ethicon) suture thread tying it 4 times followed by removing the needle. A total of fourteen mice were recruited in the ligation over needle group. Two mice were excluded immediately postoperatively, one mouse died from anesthesia while performing the procedure and four mice were excluded day three postoperatively. A total of seven mice were included in this study group (Procedure success rate 50%). One mouse was found dead (unknown cause) on day six. One mouse was found dead on day nine (injury from other mouse in cage). One mouse escaped on day twenty-four. A total of four mice completed follow up until day 42 after the procedure. The base line mean perfusion was -7.35% ± 1.85. Immediately post procedure mean perfusion was -71.87% ± 4.43. Then mean difference in perfusion remained below the base line reading on days 3, 6, 9, and 12 (-57.63% ± 9.28, -41.08% ± 11.75, -35.92% ± 16.97 and 20.38% ± 8.99 respectively). From day 15 on wards mean perfusion progressively improved remaining near base line. A series of LDPI scans from this group are presented in Figure 2A. The mean differences in perfusion between the right (ischemic) and the left (normal) plotted against time are shown in Figure 2B. Light microscopic examination of tissue sections from the right calf muscle showed loss of muscle fibers and replacement with fibrosis and adipose tissue, congested vessels were also seen; all other tissue sections did not have any abnormalities (see Figure 2C).

### Transfixation group

**Figure 3:**
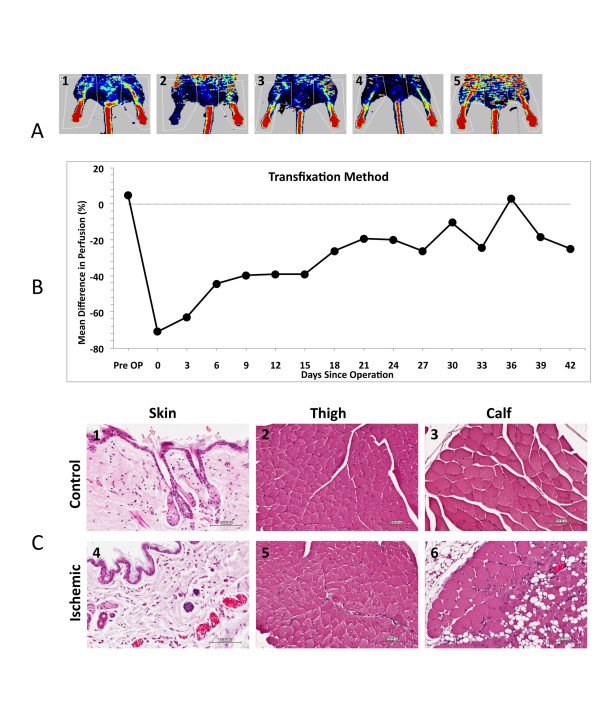
**3A; Laser Doppler perfusion Images, Transfixation group; Pre-operative, Post-Operative, Day 12, Day 24 and Day 42 post ischemia (1,2,3,4 and 5 respectively)**. 3B; Mean Difference in perfusion, Transfixation group. Figure 3C; Skin from medial aspect of left thigh with normal appearance of dermis, epidermis and skin appendages (1); Left thigh with normal appearance of skeletal muscle fibers (2); Left calf with normal appearance of skeletal muscle fibers (3); Skin from medial aspect of right thigh with mild vascular congestion and mild inflammatory cellular infiltration (4); Right thigh with normal appearance of skeletal muscle fibers (5); Right calf with vascular congestion, damage to some skeletal muscle fibers and replacement by adipose tissue (6). All sections were taken at 200 ×, stained with Hematoxylin and Eosin stain. Scale bars are 100 μm.

The transfixation method was performed by flattening the femoral artery with micro forceps followed by transfixating the approximate center with 9-0 prolene (Ethicon) and then tying it 4 times. A total of twelve mice were recruited in the transfixation group. Two mice were excluded immediately postoperatively, one mouse died during the procedure from anesthetic causes and three mice were excluded on day 3. A total of six mice were included in this group (procedure success rate 50%). One mouse was found dead due to (unknown cause) on day twenty-one. One mouse died from anesthetic complications on day thirty-three. A total of four mice completed follow up until day 42 after the procedure. The base line mean perfusion was 4.65% ± 4.05. Immediately post procedure mean perfusion was -70.82% ± 4.73. Mean perfusion improved following the procedure on days 3 and 6 (-62.95% ± 8.85 and -44.5% ± 12.38 respectively) and was followed by a plateau on days 9, 12 and 15 (-39.82% ± 12.23, -39.2% ± 10.71 and -39.1% ± 9.23 respectively). From day 15 onwards perfusion progressively improved remaining well below base line until crossing it on day 36 (-26.42% ± 12.05, -19.48% ± 7.80, -20.08% ± 15.46, -26.24% ± 15.12, -10. 46% ± 12.88, -24.48% ± 2.59 and 2.82% ± 4.86 on days 15, 18, 21 24, 27, 30, 33 and 36 respectively). A series of LDPI from this group are presented in Figure 3A. The mean differences in perfusion between the right (ischemic) and the left (normal) plotted against time are shown in Figure 3B. Light microscopic examination of tissue sections of skin from the right limb showed congested blood vessels and mild inflammatory cellular infiltrate (neutrophils, eosinophils and macrophages) in the upper dermis. Sections from the right calf had loss of muscle fibers and replacement by fibrosis and adipose tissue. All other sections did not have any abnormalities (see Figure 3C).

## Discussion

The major findings of our paper are: 1) recovery pattern of ischemia is independent of initial postoperative decrease in perfusion and 2) calf muscle is the sample of choice for obtaining representative results. The limitations of our study include: 1) relatively low success rate, 50% in both methods and 2) limited number of mice that completed the study duration, n = 4.

Previously described models of ischemia completely impede blood flow [7-11], causing an acute ischemic episode. Variations seen between the different methods employed include: use of suture material or electro-cautery [12], inclusion of the accompanying vein [13-15] and extent of excision which ranges from the external iliac artery to the popliteal vessels [16-23]. The end result of using such procedures results in an immediate post procedure drop in perfusion ranging from -90% to -75% and a perfusion ranging from - 5% to - 60% twenty eight days after the procedure [13-20]. The post procedure decrease in perfusion of the ligation over needle and transfixation methods is comparable (-71.87% and -70.82%, respectively) to previously described methods. However, when the perfusion on the 27^th ^day after the procedure is well above what can be achieved in previously described methods (-12.25% and -26.24%, ligation over needle and transfixation methods respectively).

In conclusion, both methods described were successful in inducing ischemia. The major differences between them is the duration of the ischemia postoperatively and the pattern of recovery. The optimum area to obtain skeletal muscle samples is from the calf. The currently described models do not pose major improvements over previously described methods.

## Competing interests

The authors declare that they have no competing interests.

## Authors' contributions

HA, TA and SA participated in the design, animal work, sample collection and writing of the manuscript. MAt participated in the preparation of the histological samples and data analysis. AQ, HT and PS participated in the design, data analysis and writing of the manuscript. SV, AA, BA, CN and MAl participated in the design, data interpretation and critical appraisal of the manuscript. All authors read and approved the final manuscript.
